# LncRNA PVT1 promotes the growth of HPV positive and negative cervical squamous cell carcinoma by inhibiting TGF-β1

**DOI:** 10.1186/s12935-018-0567-2

**Published:** 2018-05-08

**Authors:** Xiuli Wang, Guichan Wang, Lijuan Zhang, Jianglin Cong, Jianqing Hou, Chunyan Liu

**Affiliations:** 1 Department of Gynecology, The Affiliated Yantai Yuhuangding Hospital of Qingdao University, Yantai, 264000 Shandong People’s Republic of China; 2 Department of Obstetrics and Gynecology, The Affiliated Yantai Yuhuangding Hospital of Qingdao University, Yantai, 264000 Shandong People’s Republic of China

**Keywords:** Cervical squamous cell carcinoma, lncRNA PVT1, TGF-β1

## Abstract

**Background:**

Our study aimed to investigate the role of lncRNA PVT1 in cervical squamous cell carcinoma.

**Materials and methods:**

A total of 156 patients with cervical squamous cell carcinoma were enrolled in this study and human papillomavirus (HPV) infection was detected by highly sensitive PCR techniques. Serum levels of PVT1 in patients infected with different HPVs and healthy controls was detected by qRT-PCR and compared. Serum levels of PVT1 were also compared among patients with different sizes of tumor. ROC curve analysis was performed to evaluate the diagnostic values of serum for cervical squamous cell carcinoma. Survival curves were plotted by Kaplan–Meier method and compared to evaluate the prognostic values of serum PVT1 for this disease. Effects of PVT1 siRNA silencing and overexpression on proliferation of cervical squamous cell carcinoma cells were explored by CCK-8 assay. Western blot was performed to detect the expression of TGF-β1 after PVT1 siRNA silencing and overexpression.

**Results:**

No significant differences in serum levels of PVT1 were detected among patients infected with different HPVs and HPV-negative patients. However, serum levels of PVT1 were significantly higher in all patient groups than in healthy control group. Serum level of PVT1 increased with the increased sizes of primary tumor. Serum PVT1 accurately predicted the disease and its prognosis. PVT1 siRNA silencing inhibited the proliferation of cancer cells and reduced the expression of TGF-β1, while PVT1 overexpression played an opposite role.

**Conclusion:**

LncRNA PVT1 promotes the growth HPV positive and negative cervical squamous cell carcinoma by inhibiting TGF-β1.

## Background

At present, cervical cancer is considered to be the fourth most common type of cancer that leads to unacceptable high modality rate [1]. Cervical adenocarcinoma and cervical squamous cell carcinoma are two major subtypes of cervical cancer, and the latter one accounts for more than 80% of all cases [2]. Human papillomaviruses (HPV) infection is the most common cause of occurrence of cervical cancer [3, 4], and correlations between the incidence of cervical cancer and certain genotypes of HPV have been well established [5]. With the application of HPV infection screening and increased HPV vaccination rate, incidence of cervical squamous cell carcinoma has been reduced during 20th century, while incidence of this disease showed no further decrease during last several decades because of the existing of other risk factors other than HPV infection in the pathogenesis of cervical squamous cell carcinoma [6]. In addition, the prognosis of HPV-negative cervical cancer is usually poor [7].

Long non-coding RNA is a group of non-coding RNAs contain more than 200 nucleotides [8]. Studies in last several decades have shown that different lncRNAs have different functions in most normal biological as well as pathological processes in the human body [9]. HPV infection is related to the altered expression of certain lncRNAs [10], while differentially expression lncRNAs in cervical cancers are not reported. LncRNA PVT1 plays a role as oncogene in the development of various types of malignant tumors, such as non-small cell lung cancer [11], gastric cancer [12] and so on. However, the roles of PVT1 in cervical squamous cell carcinoma still haven’t been well studied.

In this study, expression of PVT1 in serum of patients with cervical squamous cell carcinoma was detected, and serum levels of PVT1 were compared among patients infected with different HPVs and in different stages of primary tumors. In addition, the diagnostic and prognostic values of serum PVT1 for cervical squamous cell carcinoma were analyzed. Effects of PVT1 siRNA silencing and overexpression on proliferation of cervical squamous cell carcinoma cells and expression of TGF-β1 were also explored.

### Patients

A total of 156 patients with cervical squamous cell carcinoma were enrolled from January 2009 to January 2012 in Affiliated Yantai Yuhuangding Hospital of Qingdao University. All patients were diagnosed by pathological tests and imaging examinations. Human papillomavirus (HPV) infection was detected by highly sensitive polymerase chain reaction (PCR) techniques. Among them, 21 patients were HPV negative, 47 patients were infected with HPV-16, 45 patients were infected with HPV-18, 15 patients were infected with HPV-11, 17 patients were infected with HPV-45, and 11 patients were infected with HPV-68. All patients received surgical resection, and tumor tissues and adjacent healthy tissues were collected during surgical operation. Tumor size was measured and patients were divided into for groups according to the long diameter: I (0–1 cm), II (1–3 cm), III (3–5 cm) and IV (> 5 cm). There were 23 patients in stage I, 34 in stage II, 54 in stage III and 45 in stage IV. At the same time, 39 normal healthy females with the same age distribution were also included to serve as control group. This study has been approved by the ethics committee of our hospital, and participants signed informed consent.

### Blood collection and serum preparation

Fasting blood (20 ml) was collected from each participant on the day of admission. Blood samples were kept at room temperature for 1 h, followed by centrifugation at 1500 rpm for 15 min to collect serum samples.

### Cell lines and cell culture

In this study, human cervical squamous cell carcinoma cell lines SiHa (HPV positive) and C33A (HPV negative) were included. At the same time, human normal cervical cell lines Ect1/E6E7 (HPV positive) and HCvEpC (HPV negative) were used as control group. All cell lines were purchased from ATCC (USA). All cells were culture under the conditions recommended by ATCC. Cells were harvest during logarithmic growth phase for following experiments.

### Establishment of PVT1 siRNA silencing and overexpression cell lines

PVT1 siRNA (Catalog No. AV16708, Thermo Fisher Scientific, USA) was used to establish PVT1 RNA silencing cell lines, and Silencer™ Select Negative Control No. 1 siRNA (Catalog No. 4390843, Thermo Fisher Scientific, USA) was used as a negative control. PVT1 overexpression vector was established by inserting an *Eco*RI–*Eco*RI fragment containing PVT1 cDNA into pIRSE2-EGFP vector (Clontech, Palo Alto, CA, USA). Cells were cultured over night to reach 80–90% confluent, and transfection was performed by incubating with Lipofectamine 2000 reagent (11668-019, Invitrogen, Carlsbad, USA) at 37 °C for 4 h.

### Cell proliferation assay

Cells were inoculated onto 96-well plates with 5 × 10^3^ cells per well. Cells were cultured at 37 °C for 3–5 h to reach cell adhesion, and 100 μl of DMEM medium was added. Cells were cultured at cultured at 37 °C, and 10 μl of CCK-8 was added at 24, 48, 72 and 96 h later. After incubation for another 3 h, OD values at 450 nm were measured using a microplate reader.

### Real-time quantitative reverse transcription PCR

Trizol reagent (Invitrogen, USA) was used to extract total RNA from tumor tissues, normal tissues, serum and in vitro cultured cells according the instructions. After that, cDNA was then synthesized using total RNA as template and SuperScript III Reverse Transcriptase system (Thermo Fisher Scientific, USA). PCR reaction system was prepared using cDNA and SYBR^®^ Green Real-Time PCR Master Mixes (Thermo Fisher Scientific, USA). The following primers were used: 5′-TGAGAACTGTCCTTACGTGACC-3′ (forward) and 5′-AGAGCACCAAGACTGGCTCT-3′(reverse) for PVT1; 5′-GACCTCTATGCCAACACAGT-3′ (forward) and 5′-AGTACTTGCGCTCAGGAGGA-3′ (reverse) for β-actin. PCR reaction was carried out on CFX96 Touch™ Real-Time PCR Detection System (Bio-Rad, USA). PCR reaction conditions were: 95 °C for 40 s, followed by 40 cycles of 95 °C for 12 s and 60 °C for 37 s. Ct values were processed using 2^−ΔΔCT^ method, and relative expression level of PVT1 was normalized to endogenous control β-actin.

### Western-blot

Total protein were extracted from cells using Cell lysis solutions (Thermo Fisher Scientific, USA) and quantified by BCA assay. Gel electrophoresis (10% SDS-PAGE) was performed with 30 µg protein from each sample, followed by transmembrane to PVDF membranes. Blocking was performed with 5% skimmed milk at room temperature for 2 h. After that membranes were washed and incubated with primary antibodies including rabbit anti-TGF-β1 antibody (1:2000, ab92486, Abcam) and anti-GAPDH antibody (1:1000, ab9485, Abcam) overnight at 4 °C. The next day, membranes were washed and further incubated with anti-rabbit IgG-HRP secondary antibody (1:1000, MBS435036, MyBioSource) for 2 h at room temperature. Signal detection was performed using ECL (Sigma-Aldrich, USA) method. Relative expression level of TGF-β1 was normalized to endogenous control GAPDH using Image J software.

### Statistical analysis

SPSS19.0 (SPSS Inc., USA) statistical software was used. Measurement data were expressed as (${\bar{\text{x}}} \pm {\text{s}}$), and t test was used for the comparisons between two groups, and comparisons among multiple groups were performed by one way analysis of variance, followed by LSD test. Comparisons of count data were performed using Chi square test. p < 0.05 was considered to be statistically significant.

## Results

### Serum levels of PVT1 in different patient groups and control group

**Fig. 1:**
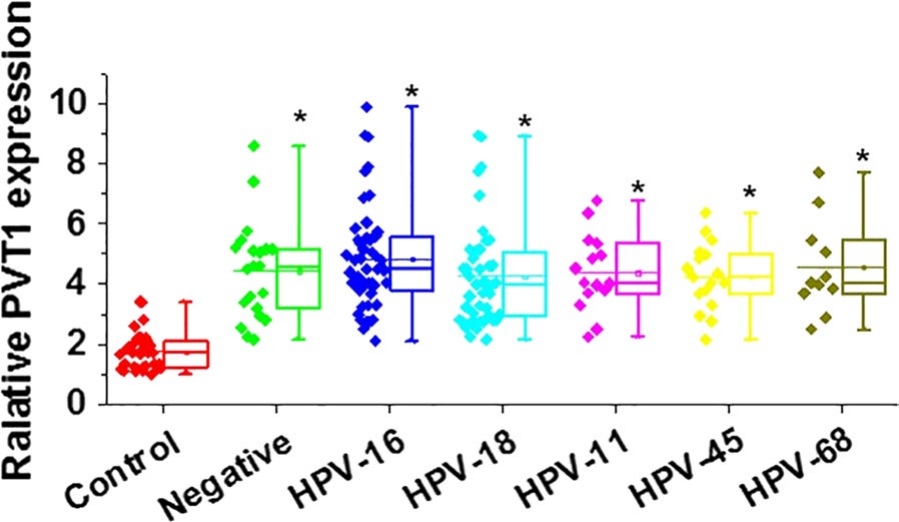
Serum levels of PVT1 in different patient groups and control group

Serum levels of PVT1 was detected by qRT-PCR and compared among different patient groups and control group. In this study, five HPV strains, including HPV-16, HPV-18, HPV-11, HPV-45 and HPV-68 were detected in tumor tissues of patients with cervical squamous cell carcinoma. As shown in Fig. 1, no significant differences in serum levels of PVT1 were detected among patients infected with different HPVs and HPV-negative patients. However, serum levels of PVT1 were significantly higher in all patient groups than in healthy control group. Those data suggest that upregulation of PVT1 may participate in the pathogenesis of cervical squamous cell carcinoma through a HPV-independent pathway.

### Comparison of serum levels of PVT1 among patients with different sizes of tumor

**Fig. 2:**
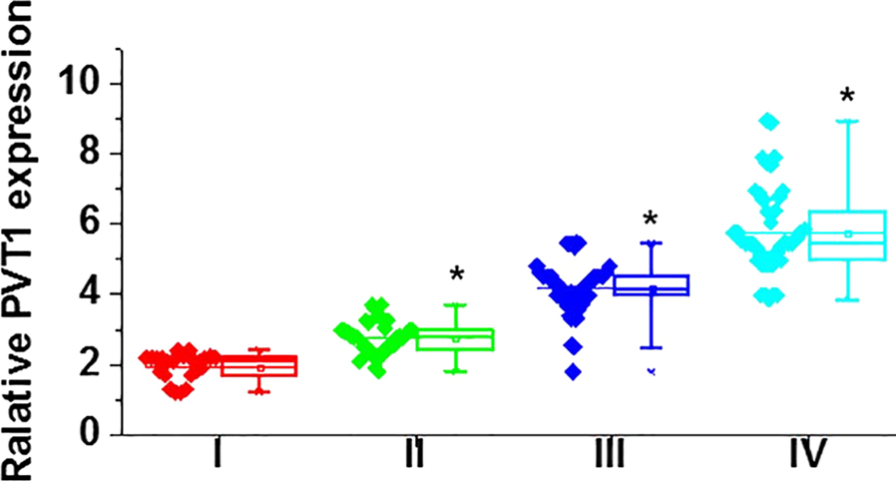
Comparison of serum levels of PVT1 among patients in different stages of primary tumor. *Compared with one stage before, p < 0.05

Our data have showed that serum levels of PVT1 were abnormally increased in patients with cervical squamous cell carcinoma. Therefore, serum levels of PVT1 may also be different in patients with different sizes of tumor. As shown in Fig. 2, serum levels of PVT1 were significantly increased with the increased sizes of cervical squamous cell carcinoma.

### Diagnostic and prognostic values of serum PVT1 for cervical squamous cell carcinoma

**Fig. 3:**
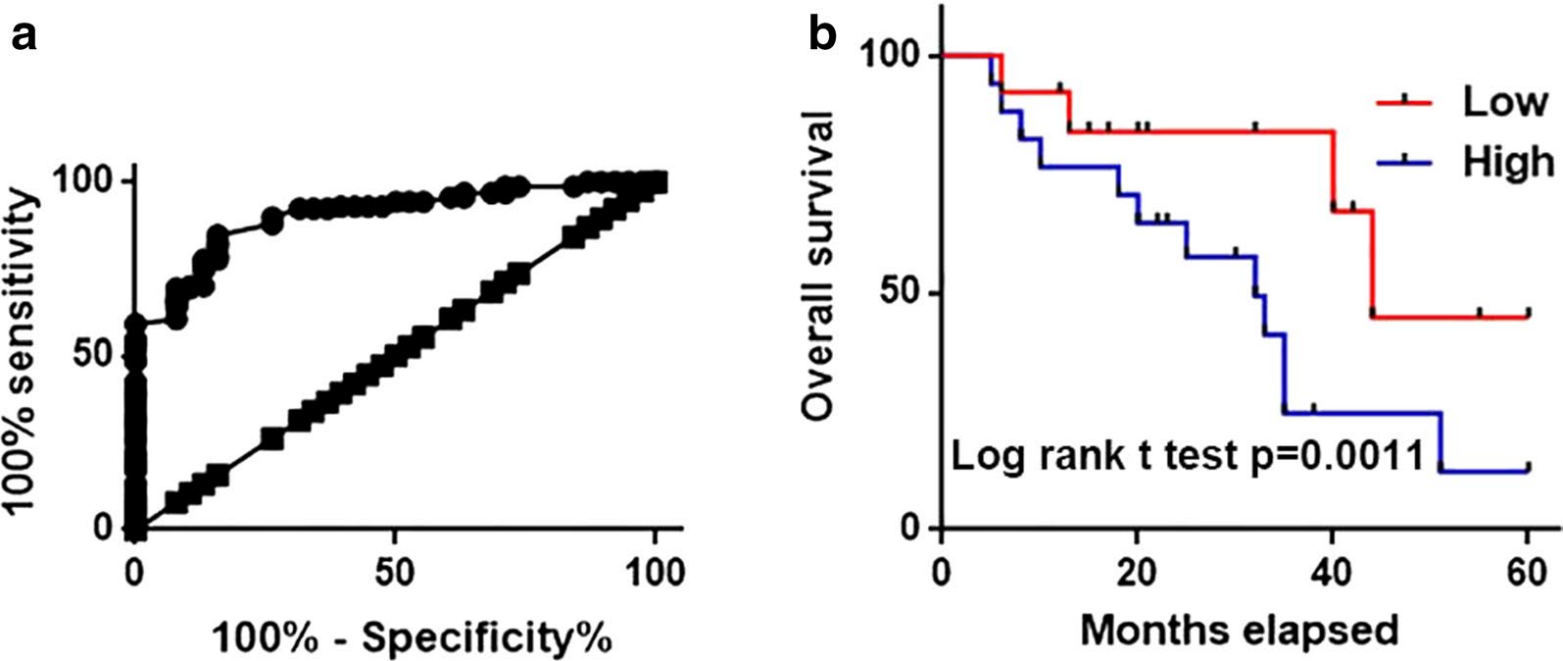
Diagnostic and prognostic values of serum PVT1 for cervical squamous cell carcinoma. **a** ROC curve analysis for the diagnostic values of serum PVT1 for cervical squamous cell carcinoma; **b** comparison of survival curves of patients with high and low serum level of PVT1

ROC curve analysis was performed to evaluate the diagnostic values of serum PVT1 for cervical squamous cell carcinoma. As shown in Fig. 3a, the area under the curve of serum PVT1 in the diagnosis was 0.9030 with 95% confident interval of 0.8567–0.9472 (p < 0.0001), indicating that serum PVT1 is an accurate biomarker for cervical squamous cell carcinoma. According to the median serum level of serum PVT1 patients were divided into high level group and low level group. Kaplan–Meier method was used to draw survival curves for both groups. Log-rank test was used to compare two survival curves. As shown in Fig. 3b, overall survival of cervical squamous cell carcinoma patient with high serum level of PVT1 was significantly shorter that that of patients with low serum level of PVT1 (p < 0.05). Those data suggest that serum level of PVT1 may serve as prognostic marker for patients with gastric cancer.

### Effects of PVT1 siRNA silencing and overexpression on proliferation of cervical squamous cell carcinoma

**Fig. 4:**
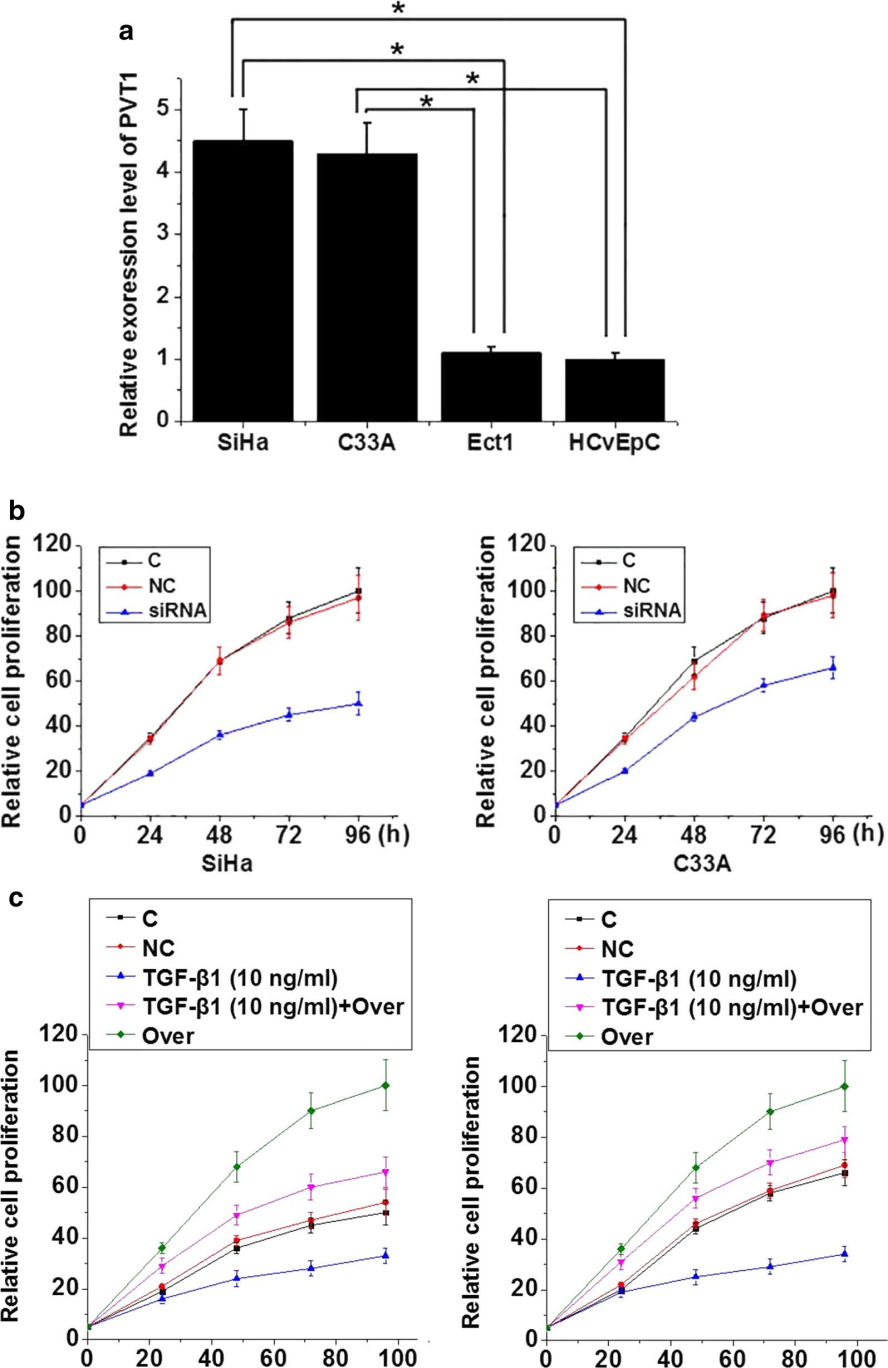
Effects of PVT1 siRNA silencing and overexpression on proliferation of cervical squamous cell carcinoma. **a** Expression of PVT1 in different cell lines; **b** Effects of PVT1 siRNA silencing on proliferation of cervical squamous cell carcinoma; c Effects of PVT1 siRNA overexpression on proliferation of cervical squamous cell carcinoma. *p < 0.05; C, control cells without transfection; NC, negative control cells transfected with negative control siRNA or empty vector; siRNA, cells transfected with PVT1 siRNA; Over, cells transfected with PVT1 expression vector

Expression of PVT1 in human cervical squamous cell carcinoma cell lines SiHa (HPV positive) and C33A (HPV negative) and normal cervical cell lines Ect1/E6E7 (HPV positive) and HCvEpC (HPV negative) was detected by qRT-PCR. As shown in Fig. 4a, expression level of PVT1 was significantly higher in two cervical squamous cell carcinoma cell lines than in two normal cells. However, no significant differences in expression level of PVT1 were found between SiHa and C33A or Ect1/E6E7 and HCvEpC, indicating that HPV infection has no significant effects on the expression of PVT1 in those cell lines. PVT1 siRNA silencing and overexpression cell lines were established to investigate the role of PVT1 in the proliferation of cervical squamous cell carcinoma cells. As shown in Fig. 4b, PVT1 siRNA silencing significantly inhibited cell proliferation of two cervical squamous cell carcinoma cell lines. In contrast, PVT1 overexpression significantly promoted cell proliferation of two cervical squamous cell carcinoma cell lines (Fig. 4c). TGF-β1 has anti-proliferative functions [13], and PVT1 can regulate the expression of TGF-β1 [14]. In this study, treatment with TGF-β1 (10 ng/ml, Sigma-Aldrich, USA) for 1 h not significantly inhibited cell proliferation of two cervical squamous cell carcinoma cell lines, but also significantly reduce the enhancing effects of PVT1 overexpression on cell proliferation. Those data suggest that expression level of PVT1 is a positive regulator of proliferation of cervical squamous cell carcinoma cells, and this function is very likely to be achieved through interactions with TGF-β1.

### Effects of PVT1 siRNA silencing and overexpression on expression of TGF-β1

**Fig. 5:**
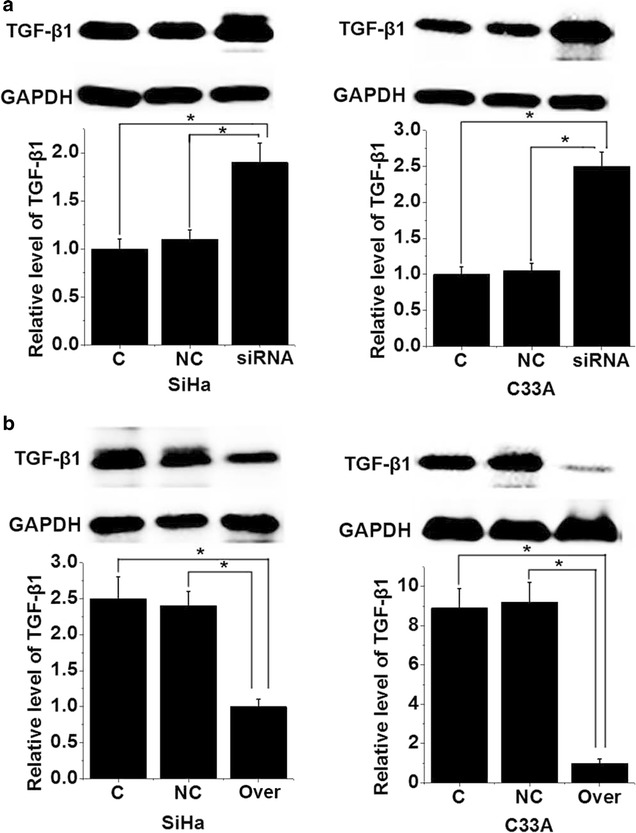
Effects of PVT1 siRNA silencing and overexpression on expression of TGF-β1. **a** Effects of PVT1 siRNA silencing on expression of TGF-β1; **b** effects of PVT1 overexpression on expression of TGF-β1. *p < 0.05; C, control cells without transfection; NC, negative control cells transfected with negative control siRNA or empty vector; siRNA, cells transfected with PVT1 siRNA; Over, cells transfected with PVT1 expression vector

To further investigated the interactions between TGF-β1 and PVT1, **e**ffects of PVT1 siRNA silencing and overexpression on expression of TGF-β1 in two cervical squamous cell carcinoma cell lines were explored. As shown in Fig. 5a, PVT1 siRNA silencing significantly increased the expression level of TGF-β1 in both cell lines (p < 0.05). In contrast, PVT1 overexpression significantly reduced the expression of TGF-β1 in two cell lines (p < 0.05). Especially in C33A cell line, expression level of PVT1 reduced by more than eight times after PVT1 overexpression. Those data suggest that PVT1 can negatively regulate the expression of TGF-β1 to participate in the growth of cervical squamous cell carcinoma.

## Discussion

Most cases of cervical squamous cell carcinoma are caused by HPV infection [3]. HPV infection is usually harmless but long-term infection of HPV may increase the risk of the occurrence of malignant tumors in various sites, such as cervix, vagina, penis, anus and oropharynx [3]. Different genotypes of HPV causes different incidences of cervical squamous cell carcinoma, and two genotypes of HPV, including HPV-16 and -18 causes about 70% cases of HPV-positive cervical squamous cell carcinoma [3]. Consistent with previous studies, in this study, 135 patients with cervical squamous cell carcinoma were proved to be HPV-positive, accounting for 86.5% of all the cases. In addition, 47 patients were infected with HPV-16 and 45 patients were infected with HPV-18, accounting for 68.4% of all the cases. HPV infection is a major but not the only cause of this disease. In this study, 21 patients were proved to be HPV-negative, accounting for 13.5% of all the cases.

LncRNA PVT1 plays a role as oncogene in the development of various types of malignant tumors and shows abnormally upregulated expression. In the study of pancreatic cancer, Huang et al. found that expression level of the lncRNA PVT1 was significantly increased in cancer patients than in normal healthy controls, and the upregulated expression level of PVT1 was significantly correlated with poor prognosis of those patients [15]. In another study, expression level of PVT1 was also found to be abnormally increased in gastric cancer patients, and abnormal expression of PVT1 showed promising diagnostic and prognostic values for this disease [16]. Previous studies also confirmed that PVT1 is upregulated in cervical cancer and promote the progression of disease [17–19]. Consistent with previous studies, in this study, expression level of PVT1 was found to be significantly higher in cervical squamous cell carcinoma patients than in normal controls. In addition, expression level of PVT1 was increased with the increased stage of primary tumor. HPV infection can induce the abnormal expression of certain lncRNAs [10]. In our study, no significant differences in serum levels of PVT1 were detected among patients infected with different HPVs and HPV-negative patients. Those data suggest that upregulated expression of PVT1 may participate in the pathogenesis of cervical squamous cell carcinoma through a HPV infection-independent pathway. Besides that, serum PVT1 also showed promising diagnostic and prognostic values for cervical squamous cell carcinoma. Therefore, PVT1 may serve as a target to improve the treatment outcomes and prognosis of those patients. It is worth to note that several lncRNAs, such as GAS5 [20] and HOTAIR [21] show clinical values for the diagnosis and prognosis of cervical cancers. Combination of multiple biomarkers may improve the diagnosis.

Previous studies have shown that PVT1 can promote the proliferation of various types of cancer cells, such as hepatocellular carcinoma cells [22], thyroid cancer cells [23] and so on. PVT1 siRNA silencing inhibited the proliferation of two cervical squamous cell carcinoma cell lines, while PVT1 overexpression promoted the proliferation of cancer cells, indicating that PVT1 expression may promote the growth of cervical squamous cell carcinoma by promoting the proliferation of cancer cells. TGF-β1 plays different roles in the development and progression of tumors. TGF-β1 not only inhibits the growth of tumors through its anti-proliferative functions [13], but also promotes tumor metastasis by inducing epithelial–mesenchymal transition [24]. Expression of TGF-β1 was regulated by PVT1 [14]. Therefore, it will be reasonable to hypothesize that PVT1 may regulate TGF-β1 to participate in cervical squamous cell carcinoma. In this study, PVT1 siRNA silencing significantly increased the expression level of TGF-β1. In contrast, PVT1 overexpression significantly reduced the expression of TGF-β1. Those data suggest that PVT1 can negatively regulate the expression of TGF-β1 to participate in the growth of cervical squamous cell carcinoma. However, the molecular mechanism of the regulation of TGF-β1 by PVT1 is still unknown. Therefore, further studies are still needed.

## Conclusion

In conclusion, expression of PVT1 in patients with cervical squamous cell carcinoma was not affected by HPV infection. Serum levels of PVT1 were significantly higher in cervical squamous cell carcinoma patients than in healthy controls. Serum level of PVT1 increased with the increased size of primary tumor. Serum PVT1 can serve as a promising diagnostic and prognostic biomarker for cervical squamous cell carcinoma PVT1 siRNA silencing inhibited the proliferation of cancer cells and reduced the expression of TGF-β1, while PVT1 overexpression played an opposite role. So we conclude that lncRNA PVT1 can promote the growth HPV positive and negative cervical squamous cell carcinoma by inhibiting TGF-β1. Our study is limited by the small sample size. Future studies with bigger sample size are needed to further confirm the conclusions in the present study.
